# Concomitant training in robotic and laparoscopic liver resections of low-to-intermediate difficulty score: a retrospective analysis of the learning curve

**DOI:** 10.1038/s41598-024-54253-z

**Published:** 2024-02-13

**Authors:** Lorenzo Bernardi, Emanuele Balzano, Raffaello Roesel, Davide Ghinolfi, Filippo Vagelli, Giacomo Menconi, Antonietta Petrusic, Francesco Mongelli, Pietro Majno-Hurst, Paolo De Simone, Alessandra Cristaudi

**Affiliations:** 1 Department of Surgery, Ente Ospedaliero Cantonale (EOC), Lugano, Switzerland; 2 Hepato-Biliary Surgery and Liver Transplant Division, Azienda Ospedaliera Universitaria Pisana (AOUP), University of Pisa, Via Paradisa, 2, 56124 Pisa, Italy; 3 Faculty of Biomedical Sciences, University of Southern Switzerland (USI), Lugano, Switzerland; 4 Department of Surgical, Medical, Biochemical Pathology and Intensive Care, University of Pisa, Pisa, Italy

**Keywords:** Outcomes research, Liver

## Abstract

In the setting of minimally invasive liver surgery (MILS), training in robotic liver resections (RLR) usually follows previous experience in laparoscopic liver resections (LLR). The aim of our study was to assess the learning curve of RLR in case of concomitant training with LLR. We analyzed consecutive RLRs and LLRs by a surgeon trained simultaneously in both techniques (Surg1); while a second surgeon trained only in LLRs was used as control (Surg2). A regression model was used to adjust for confounders and a Cumulative Sum (CUSUM) analysis was carried out to assess the learning phases according to operative time and difficulty of the procedures (IWATE score). Two-hundred-forty-five procedures were identified (RobSurg1, n = 75, LapSurg1, n = 102, LapSurg2, n = 68). Mean IWATE was 4.0, 4.3 and 5.8 (p < 0.001) in each group. The CUSUM analysis of the adjusted operative times estimated the learning phase in 40 cases (RobSurg1), 40 cases (LapSurg1), 48 cases (LapSurg2); for IWATE score it was 38 cases (RobSurg1), 33 cases (LapSurg1), 38 cases (LapSurg2) respectively. Our preliminary experience showed a similar learning curve of 40 cases for low and intermediate difficulty RLR and LLR. Concomitant training in both techniques was safe and may be a practical option for starting a MILS program.

## Introduction

Minimally invasive liver surgery (MILS) relies on two approaches: laparoscopic liver resections (LLR) and robotic liver resections (RLR). While LLR started in the early nineties and diffused homogeneously in the following 20 years, RLR spread later in the early years 2000, and was implemented more slowly^1–3^.

The slower adoption of robotics may be explained by the higher costs, the limited access to the robotic platforms in several institutions, and the lack of specific robotic instruments for hepatic parenchymal transection, like the Cavitron Ultrasonic Surgical Aspirator (CUSA) which is only available for LLR^3–5^. Parenchymal transection in RLR is usually performed with the robotic instruments (mostly bipolar devices) by the “clamp-crush” technique or by having the assistant surgeon at the operative table handling the CUSA, in what is in fact a hybrid (laparoscopic and robotic) procedure^5,6^. Furthermore, a possible superiority of robotics over laparoscopy in liver resections is still matter of debate; a potential advantage of the robotic approach is reported for the most difficult resections and for those requiring complex biliary or vascular reconstructions which nowadays are still mostly performed by open surgery^7–9^.

Because of the earlier diffusion of LLR, training in RLR usually follows previous experience in LLR^10,11^. Only few centers introduced RLR from the beginning, or both LLR and RLR concomitantly, showing the feasibility and safety of the simultaneous implementation of the two programs^12^. Accordingly, the process needed to acquire proficiency in RLR has been investigated mostly among surgeons already experienced in LLR, and the safe and shorter learning phase for RLR than for LLR may result from the previous LLR experience of the surgeons^13–16^.

The description of the learning curve of RLR in case of concomitant implementation of both MILS techniques, and the demonstration of the feasibility and safety of the simultaneous training, is particularly relevant now that a new generation of surgeons can access to RLR from the start. It is currently unclear whether beginners in MILS, should receive prior LLR training before embarking in RLR, could receive simultaneous training in both techniques, or could be trained directly from open to RLR.

The objective of this study was to assess the learning curve of RLR for a surgeon who started concomitantly RLR and LLR training, comparing it to the learning curve in a surgeon who only did LLR.

## Methods

### Ethical approval and informed consent statement

The study was approved by the local ethics committee of both institutions participating to the study (Institutional Review Board of the University Hospital of Pisa, and Comitato etico cantonale del Ticino). All participants (or their legal guardians) have signed an informed consent (patients from Pisa center) or received a non-objection letter (patients from Lugano center) before inclusion in the study. All methods were performed in accordance with the relevant guidelines and regulations. This study has been performed in accordance with the Declaration of Helsinki.

#### Population and design of the study

This is a retrospective study including patients who underwent MILS (either robotic or laparoscopic) in two referral centers for hepatobiliary surgery from May 2014 to November 2022: the Hepato-biliary surgery and liver transplant unit, University of Pisa, Italy, and the Hepato-pancreatic and biliary (HPB) surgery unit of the Ente Ospedaliero Cantonale, Lugano, Switzerland.

Data were extracted from the prospective institutional databases and included clinical characteristics, intra- and postoperative outcomes. Morbidity was classified according to Clavien-Dindo^17^. In patients receiving multiple liver resections, the IWATE score was obtained from the most difficult one; in case of lesions in more than one liver segment, the IWATE score was calculated on the most difficult segment^18^.

Patients were considered for inclusion only if operated by one of two senior hepatobiliary surgeons: EB (Center 1, Pisa), and AC (Center 2, Lugano):

(1) Surgeon 1 (EB) was a senior hepatobiliary surgeon under simultaneous training in both robotic and laparoscopic liver surgery, in a center (center 1) where both MILS techniques were implemented at the same time. In this center, MILS is performed with a concomitant liver transplant activity (approximately 150 transplants/year). The training in robotic liver surgery was initially limited to this surgeon, while laparoscopic procedures were performed by all senior members of the team. Previous surgical experience of Surgeon 1 consisted of approximately 150 liver transplants, 80 open liver resections, 100 procedures of general surgery (open and laparoscopic). The MILS procedures from the surgeon 1 (robotic and laparoscopic) were performed in the period May 2014 to November 2022. The first 10 robotic procedures were tutored by a senior surgeon (proctor) from an external institution. No external mentoring was performed for LLR. Cases for robotic or laparoscopic technique were selected to be similar as per patients and tumors characteristics (i.e., IWATE score), starting with superficial lesions located in antero-lateral liver segments at the beginning of the experience, then progressively scaling up with the difficulty (i.e., posterior location, close relation with vascular or biliary structures). While the allocation to robotics depended on the availability of the robotic platform (1 session every one or two weeks, stable during the study period), laparoscopy was freely available up to 2 days per week. The details of the implementation of the MILS program in this center were described previously^12^.

(2) Surgeon 2 (AC) was a senior hepatobiliary surgeon under training only in LLR. Surgeon 2 was mentored for approximately two years by another senior surgeon from the same institution already expert in open and laparoscopic liver surgery. Previous experience consisted of 50 open liver procedures and more than 200 general surgery procedures (open and laparoscopic). The LLR of Surgeon 2 were used as external control group for the training in MILS of surgeon 1. The laparoscopic procedures from surgeon 2 were performed in the period May 2018 to November 2022. For surgeon 2, laparoscopy was available 2–3 times per week.

Procedures performed by other surgeons of both teams, cyst’s fenestrations, hand-assisted procedures, non-elective procedures, and intraoperative switch of strategy (switch from MILS to local radiological procedures, i.e., local thermal ablation) were excluded from the analysis.

The selection policy of patients to MILS in both centers was reported elsewhere in detail^19^. Briefly, MILS was initially adopted in the case of single tumors with favorable location (i.e., subcapsular, anterolateral segments), then with the increasing of experience, it was progressively introduced for multiple tumors (generally up to 3) located in posterior or superior segments. Both MILS techniques were adopted in normal livers or in the setting of compensated cirrhosis (i.e., Child A or B) with or without signs of portal hypertension.

Patients and relative procedures were divided into three groups according to the operating surgeon and the technique used (RobSurg1: robotic procedures by EB; LapSurg1: laparoscopic procedures by EB; LapSurg2: laparoscopic procedures by AC).

The learning curve was measured according to 4 variables: adjusted operative time, conversions to open surgery, post-operative morbidity, and difficulty of the procedures (IWATE score). We defined the learning phase as the number of cases necessary to reach a significant improvement of the outcome of interest (e.g., adjusted operative time, conversions to open surgery, post-operative morbidity, increase in the IWATE difficulty score). For the estimation of the learning curve, we used as primary endpoint the adjusted operative time; as secondary endpoints the conversions to open surgery, the post-operative morbidity, and difficulty of the procedures (IWATE score).

The feasibility and safety of the simultaneous training in both MILS techniques were surrogated by the following surgical outcomes: 90-days overall and major morbidity, conversions to open surgery, R0 rate. Strengthening the reporting of observational studies in epidemiology (STROBE) guidelines were followed^20^.

### Statistical analysis

Descriptive statistics were presented as frequencies for categorical variables and as mean with standard deviation (SD) for continuous variables. The chi-square test was used for the comparison of dichotomous variables, while continuous variables were compared with the ANOVA test. An analysis of covariance (ANCOVA) considering age, gender, difficulty of MILS (IWATE score), ASA score, previous liver surgery, previous loco-regional treatment, portal hypertension and extended resection to neighbor organs was used to estimate the adjusted operative times. A cumulative sum control chart (CUSUM analysis) was carried out for each surgeon to monitor changes in the adjusted operative time, IWATE difficulty score, conversions to open surgery, and post-operative morbidity. The CUSUM analysis is a statistical method used to analyze the deviations from an expected value in a series of consecutive observations, the reference of which is constructed on the basis (the cumulative summation) of all observations. Although statistically more robust, the CUSUM plot can be regarded as a deviation of the variable of interest (i.e., adjusted operative time, conversion, IWATE score, etc.) from the expected value for each observation (case). The two primary curves in a CUSUM analysis graph are the positive CUSUM and negative CUSUM. The first one represents the cumulative sum of positive differences between observed values and the reference value. An increasing positive CUSUM indicates that the process is yielding results above the reference value. When the positive CUSUM surpasses a predefined threshold, it indicates a particularly relevant change in the process. The negative CUSUM curve represents the cumulative sum of negative differences between observed values and the reference value. An increasing negative CUSUM indicates that the process is producing results below the reference value. Similar to the positive, when the negative CUSUM surpasses a predefined threshold, this points to a relevant change in the process. The combination of these curves visually depicts the process behaviour over time. In our study, a curve of observed values persistently below (in the case of operative time) or above (in the case of the difficulty score) the expected value marks the completion of the learning curve. The level of significance was set at 5%. All analyses were conducted with MedCalc® Statistical Software version 20.115 (MedCalc Software Ltd, Ostend, Belgium; https://www.medcalc.org; 2022).

### Surgical technique

#### Laparoscopic approach

Patients were placed in the French position (supine, legs abducted) or left semi-lateral decubitus position (for resection involving the posterior-superior liver segments). The surgeon operated in-between the legs in most of the cases or standing at the right of the patient (i.e., resection involving the posterior-superior liver segments). Four to five operative ports were placed along a J-shaped line or reversed L-shaped laparotomy incision, to be used in case of conversion. Parenchymal transection was performed by laparoscopic CUSA in both the institutions. Pringle's maneuver was used according to surgeon’s preference.

#### Robotic approach

The robotic procedures were performed using the DaVinci Si for few cases at the beginning of the experience and then with DaVinci Xi robotic platform (Intuitive) as following. In case of resection involving the antero-lateral liver segments the patient was placed in supine position. A total of 4 or 5 ports were used: three-to-four 8 mm ports for the robotic instruments (monopolar scissors, bipolar forceps, or Maryland bipolar forceps, prograsp, robotic vessel sealer) and the camera in a transversal linear fashion; one 11 mm assistance port in umbilical (resections in segments 2 and 3) or left para-umbilical position (resections in segment 4). In case of resections in the posterior-superior segments or in the right liver, we used three-to-four 8 mm ports for robotic instruments and camera in a right subcostal position, together with one 11 mm assistance port along the right pararectal line (resections in segments 6 and 7) or in umbilical position (resections in segments 5 and 8). Parenchymal transection was performed by the clamp-crush technique; laparoscopic-assistance with CUSA was not used in robotic procedures. Pringle's maneuver was applied according to surgeon’s preference.

For both LLR and RLR, intra-operative ultrasound (IO-US) was systematically used to assess the anatomy of the liver, the relation between the tumors and intra-hepatic vessels or biliary structures, and the resection margins.

## Results

**Figure 1:**
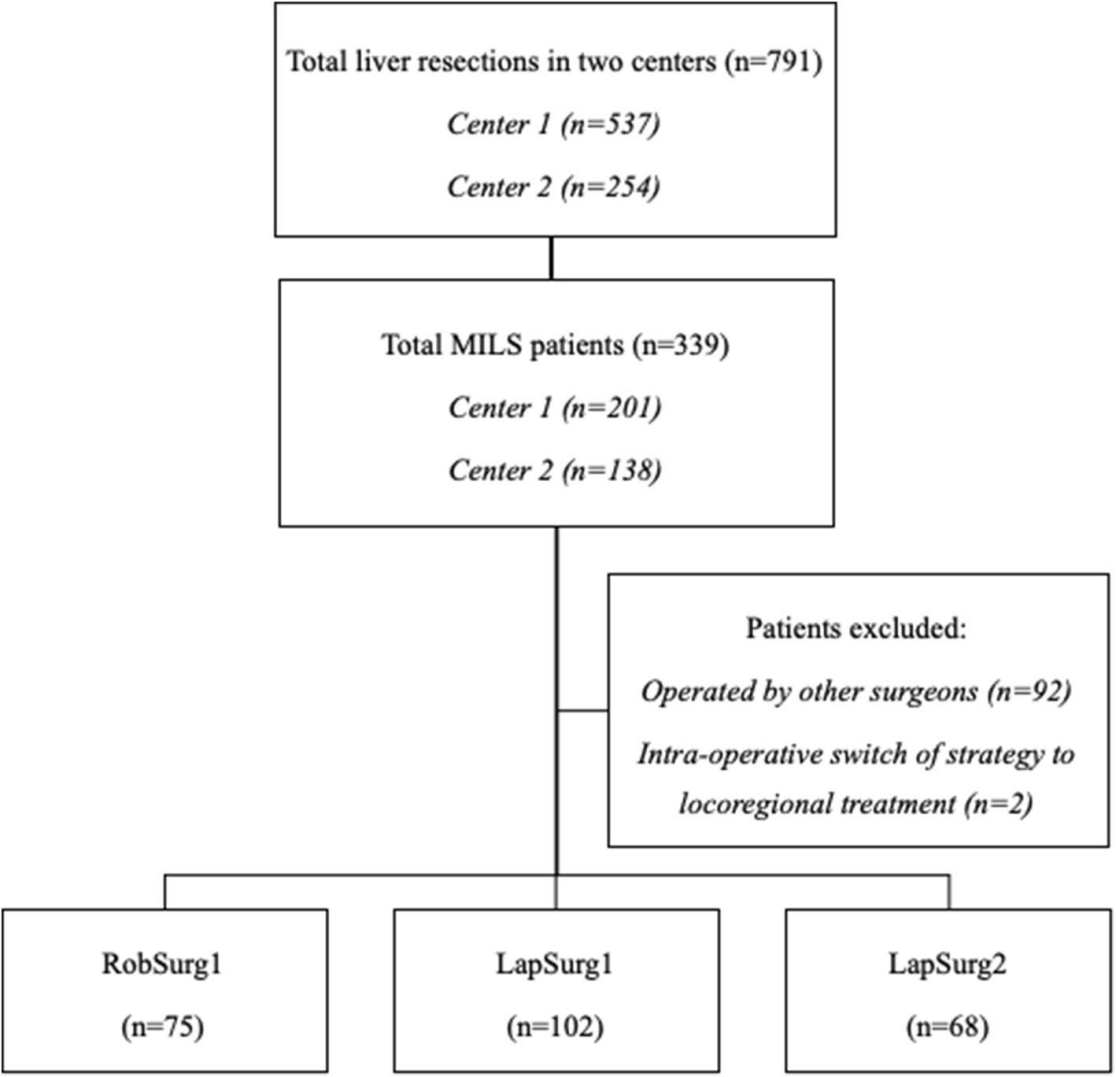
Flowchart of the study, indicating the selection process of patients/procedures analyzed.

During the study period, out of 791 patients who underwent liver resection in the two institutions (Center 1 = 537; Center 2 = 254), 339 patients were operated by MILS (RLR = 79; LLR = 260). Ninety-two MILS patients were excluded because operated by other surgeons (RLR = 2; LLR = 90), 2 patients (robotic group) because of intra-operative switch of strategy to local ablation. Two-hundred-forty-five procedures were identified and included in the analysis (RobSurg1, n = 75, LapSurg1, n = 102, LapSurg2, n = 68). The selection process is outlined in Fig. 1.

### Baseline characteristics

Baseline characteristics are detailed in Table 1. Significant differences were noted in the rates of previous abdominal surgery, while the rates of previous liver surgery were similar. Underlying chronic liver disease was more frequent in RobSurg1 (85.3%) and LapSurg1 (65.7%) groups compared to LapSurg2 (35.4%) (p < 0.001); accordingly, portal hypertension was more frequent in the group of patients operated by the surgeon 1 (RobSurg1: 33.3% and LapSurg1: 19.6%) compared to the control group (LapSurg2: 4.4%) (p < 0.001).

**Table 1:** Demographic and clinical characteristics.

Baseline	RobSurg1 n = 75	LapSurg1 n = 102	LapSurg2 n = 68	p
Age (years), mean (SD)	65.7 (12.4)	65.1 (12.3)	67.9 (10.7)	0.299
Gender (male), n (%)	48 (64.0)	67 (65.7)	34 (50.0)	0.100
BMI (kg/m^2^), mean (SD)	25.8 (4.2)	25.5 (4.6)	25.2 (4.1)	0.097
ASA score				
I, n (%)	4 (5.3)	5 (4.9)	3 (4.4)	0.268
II, n (%)	21 (28.0)	20 (19.6)	23 (33.8)	
III, n (%)	43 (57.3)	72 (70.6)	40 (58.8)	
IV, n (%)	7 (9.3)	5 (4.9)	2 (2.9)	
Previous abdominal surgery, n (%)	40 (53.3)	44 (43.1)	51 (75.0)	** < 0.001**
Previous local treatment, n (%)	5 (6.7)	16 (15.7)	16 (23.5)	**0.019**
Previous liver surgery, n (%)	7 (9.3)	6 (5.9)	10 (14.7)	0.218
Presence of comorbidities, n (%)	57 (76.0)	73 (71.6)	56 (82.4)	0.273
Cardiac disease, n (%)	19 (25.3)	28 (27.5)	25 (36.8)	0.278
Diabetes mellitus, n (%)	19 (25.3)	30 (29.4)	14 (20.6)	0.434
CRF, n (%)	5 (6.7)	6 (5.9)	7 (10.3)	0.538
Stroke, n (%)	5 (6.7)	1 (1.0)	5 (7.4)	0.080
Asthma, n (%)	2 (2.7)	5 (4.9)	0	0.170
COPD, n (%)	10 (13.3)	14 (13.7)	4 (5.9)	0.238
Chronic liver disease, n (%)	64 (85.3)	67 (65.7)	24 (35.3)	** < 0.001**
Portal hypertension, n (%)	25 (33.3)	20 (19.6)	3 (4.4)	** < 0.001**

Demographic and clinical characteristics. Dichotomous variables are expressed as number with percentage. Continuous variables are expressed as mean with standard deviation (SD).

Significant values are given in bold.

### Intra- and post-operative results of MILS

**Figure 2:**
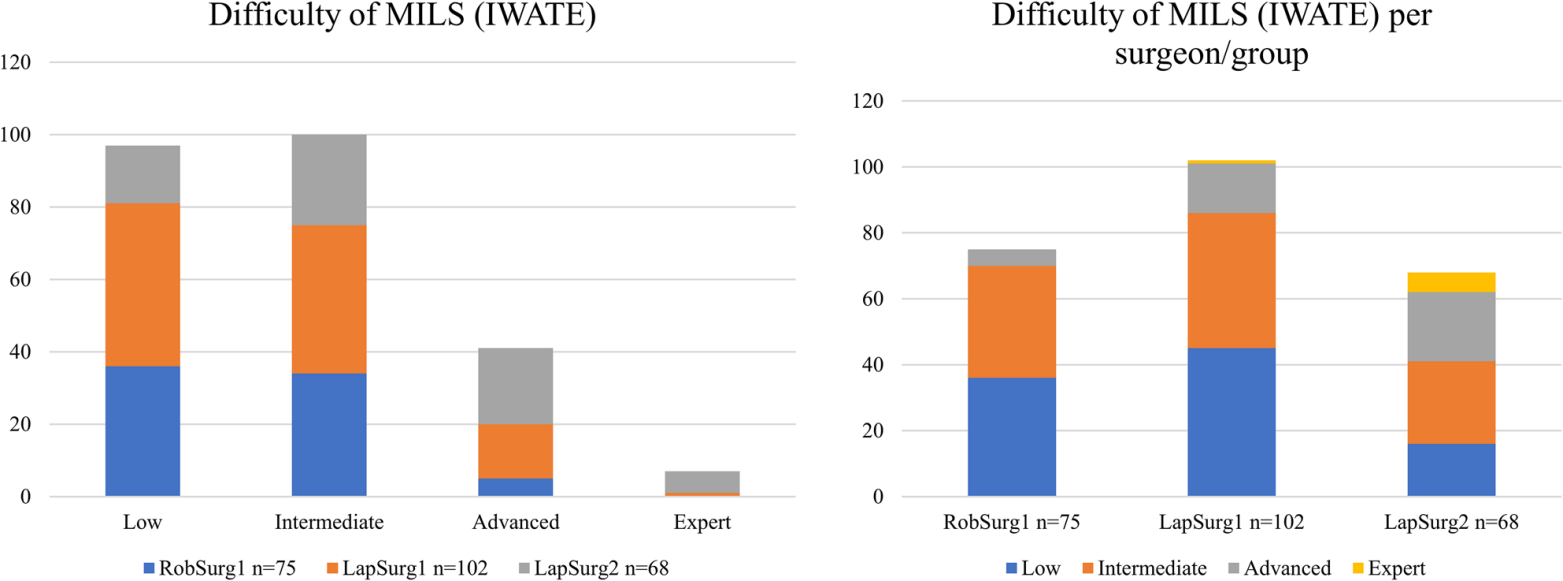
Difficulty of minimally invasive liver surgery (MILS). (**A**) The distribution of all the procedures included stratified as per category of difficulty (IWATE). (**B**) The stratification of MILS according to the level of difficulty (IWATE) in each group.

The details of the procedures and of the intra- and post-operative results of MILS in the three groups are showed in Tables 2 and 3. The mean (SD) IWATE score of MILS was 4.0 (2.0), 4.3 (2.1) and 5.8 (2.7) (p < 0.001) in RobSurg1, LapSurg1 and LapSurg2 group respectively, with the LapSurg2 group consisting of significantly more difficult liver resections. In particular, the procedures were mostly low-intermediate difficulty resections according to the IWATE score: in the RobSurg1 group there were 93.3% of low-intermediate difficulty resections (equally distributed), 6.7% of advanced resections and no expert resection. In the LapSurg1 group there were 14.7% of advanced resections and only 1 (1%) expert resections. In the LapSurg2 there were 21 (30.2%) advanced and 6 (8.8%) expert resections. The distribution of MILS as per IWATE score in each group is shown in Fig. 2. Conversion rate to open surgery was similar in the three groups (RobSurg1: 6.7%; LapSurg1: 10.8%; LapSurg2: 5.9%; p = 0.442). Mean operative time (SD) was 227 (80) min for RobSurg1, 248 (99) min for LapSurg1 and 329 (165) min for LapSurg2, being significantly longer in the latter group (p < 0.001). The mean length of hospital stay was 6 days in all the three groups (p = 0.927). Post-operative morbidity was also similar in the three groups (RobSurg1: 17.3%; LapSurg1: 22.5%; LapSurg2: 20.6%; p = 0.220).

**Table 2:** Intraoperative outcomes.

	RobSurg1 n = 75	LapSurg1 n = 102	LapSurg2 n = 68	p
IWATE difficulty score, mean (SD)	4.0 (2.0)^†^	4.3 (2.1)^¥^	5.8 (2.7)^†¥^	** < 0.001**
IWATE class of difficulty, n (%)				
Low	36 (48.0)	45 (44.1)	16 (23.5)	**0.0003**
Intermediate	34 (45.3)	41 (40.2)	25 (36.8)	
Advanced	5 (6.7)	15 (14.7)	21 (30.2)	
Expert	0 (0)	1 (1.0)	6 (8.8)	
Type of resection, n (%)				
Wedge resection	59 (79.7)	66 (64.7)	48 (70.6)	0.188
Wedge resection × 2	6 (8.0)	2 (2.0)	2 (2.9)	
Bisegmentectomy	4 (5.3)	7 (6.9)	6 (8.8)	
Bisegmentectomy + segmentectomy	0	1 (1.0)	0	
Bisegmentectomy + wedge resection	0	1 (1.0)	2 (2.9)	
Left hepatectomy	0	2 (2.0)	0	
Right hepatectomy	0	1 (1.0)	1 (1.5)	
Segmentectomy	3 (4.0)	13 (12.7)	6 (8.8)	
Segmentectomy + wedge resection	0	1 (1.0)	1 (1.5)	
Subsegmentectomy	3 (4.0)	8 (7.8)	1 (1.5)	
Trisegmentectomy	0	0	1 (1.5)	
Conversion to open surgery, n (%)	5 (6.7)	11 (10.8)	4 (5.9)	0.442
Operative time, min (SD)	227 (80)^†^	248 (99)^¥^	329 (165)^†¥^	** < 0.001**
Pringle manoeuvre, n (%)	16 (21.3)	47 (46.1)	59 (86.8)	** < 0.001**
Number of lesions, n (%)				
1	65 (86.7)	85 (84.2)	56 (83.6)	0.070
2	10 (13.3)	12 (11.9)	3 (4.5)	
≥ 3	0	4 (4.0)	8 (12.0)	
Tumour size, mm (SD)	27 (13)^†^	35 (25)	39 (41)^†^	**0.031**
R0 resection margins, n (%)	69 (92.0)	100 (98.0)	64 (94.1)	0.167

Dichotomous variables are expressed as number with percentage. Continuous variables are expressed as mean with standard deviation (SD).

Significant values are given in bold. †¥ indicate results showing statistically significant difference.

**Table 3:** Postoperative outcomes.

	RobSurg1 n = 75	LapSurg1 n = 102	LapSurg2 n = 68	p
Length of hospital stay, days (SD)	6.1 (2.5)	6.3 (2.9)	6.2 (3.5)	0.927
Patients transfused red cells, n (%)	3 (4.0)	9 (8.8)	1 (1.5)	0.093
Bleeding, n (%)	3 (4.0)	2 (2.0)	0	0.253
Bile leak, n (%)	0	5 (4.9)	3 (4.5)	0.165
Complications (Clavien-Dindo), n (%)				
I	0	9 (8.8)	5 (7.4)	0.220
II	11 (14.7)	9 (8.8)	4 (5.9)	
IIIa	1 (1.3)	3 (2.9)	3 (4.4)	
IIIb	1 (1.3)	1 (1.0)	2 (2.9)	
IV	0	1 (1.0)	0	

Dichotomous variables are expressed as number with percentage. Continuous variables are expressed as mean with standard deviation (SD).

### Learning curve (CUSUM analysis)

**Figure 3:**
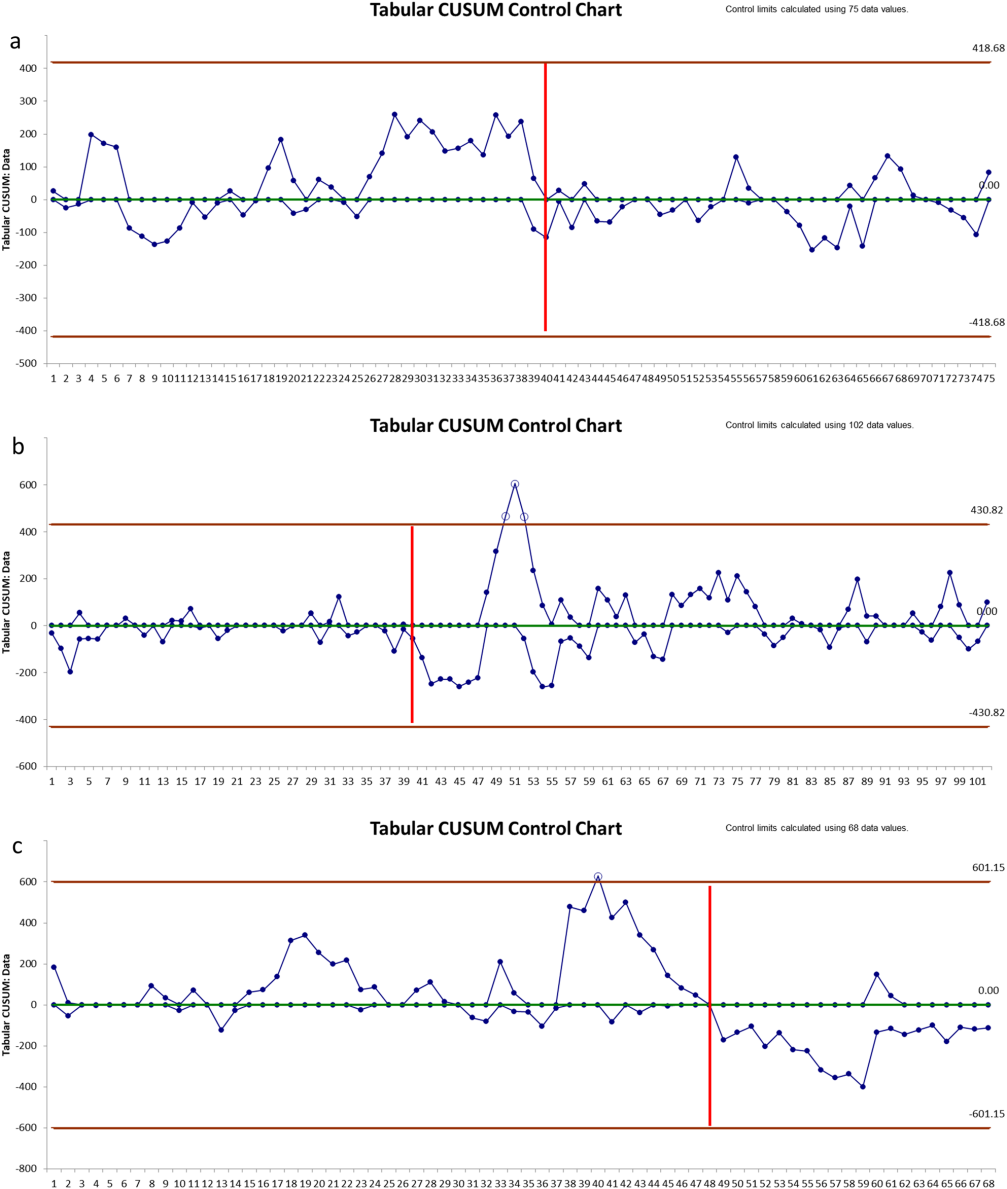
The CUSUM analysis of the adjusted operative time: (**a**) RobSurg1; (**b**) LapSurg1; (**c**) LapSurg2. The CUSUM analysis of the adjusted operative time showed a similar learning phase for RLR and LLR of 40 cases in the surgeon trained simultaneously in both MILS techniques (Surgeon 1: RobSur1 and LapSurg1). In the control group (LapSurg2), the learning phase of the adjusted operative time was 48 cases. The y-axis represents the operative time (min), the x-axis contains hepatectomies in chronological order. The red vertical line highlights the end of the learning phase.

**Figure 4:**
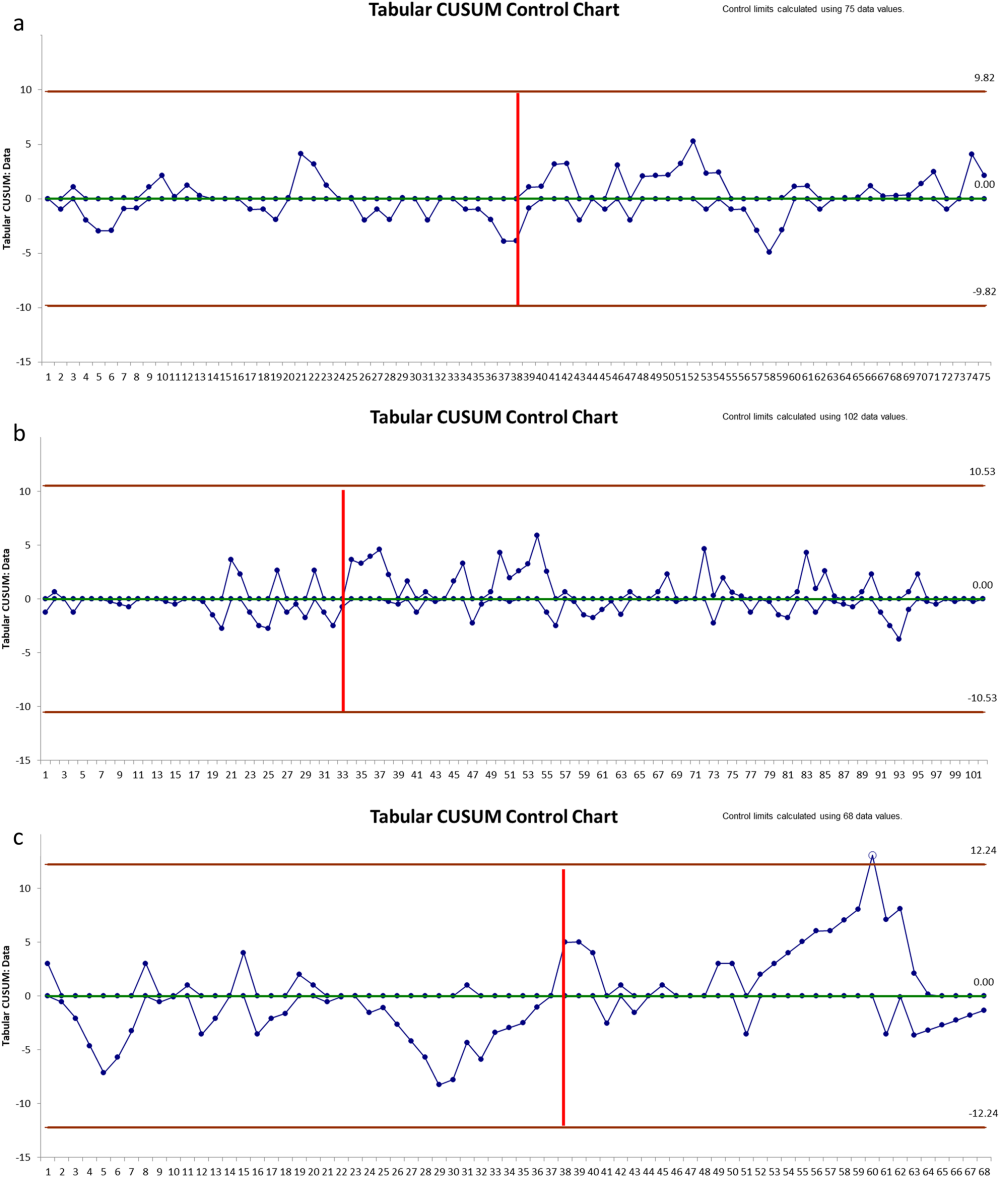
The CUSUM analysis of the IWATE criteria: (**a**) RobSurg1; (**b**) LapSurg1; (**c**) LapSurg2. The CUSUM analysis of the IWATE criteria showed a learning phase of 38 cases for RLR (RobSurg1) and of 33 cases for LLR (LapSurg1) by the surgeon 1 trained simultaneously in both MILS techniques. The learning phase was of 38 cases in the control group (surgeon 2, LapSurg2). The y-axis represents the IWATE score, the x-axis contains hepatectomies in chronological order. The red vertical line highlights the end of the learning phase.

According to the CUSUM analysis of the adjusted operative times, the learning phase was estimated in 40 cases (RobSurg1), 40 cases (LapSurg1), 48 cases (LapSurg2) (Fig. 3). The CUSUM analysis of the IWATE score showed a learning phase of 38 cases (RobSurg1), 33 cases (LapSurg1), 38 cases (LapSurg2) respectively (Fig. 4). Conversions to open surgery were uniformly distributed over the study period in all the groups, therefore, no CUSUM analysis was performed. Post-operative morbidity plateaued after 42 cases for RLRs (Fig. S1, supplementary material). Such analysis for LLRs wasn’t reliable as most of the complications occurred in the late phase, where surgeons performed the most demanding cases.

A comparative analysis of surgical outcomes (operative time, IWATE score, conversions rates and post-operative morbidity) was carried out for the learning phases vs the post-learning phases respectively, defined as detailed above, according to the CUSUM analysis of the adjusted operative time and the IWATE criteria (Table 4).

**Table 4:** Intra- and post-operative outcomes of MILS, learning phase and post-learning phase of the adjusted operative time (a) and IWATE score (b).

(a) Adjusted OT	Learning phase	Post-learning	p	Learning phase	Post-learning	p	Learning phase	Post-learning	p
	n = 40	n = 35		n = 40	n = 62		n = 48	n = 20	
Overall morbidity, n (%)	10 (25.0)	3 (8.6)	0.062	11 (27.5)	12 (19.4)	0.339	8 (16.7)	6 (30.0)	0.219
Major morbidity (CD ≥ 3), n (%)	2 (5.0)	0	0.183	1 (2.5)	4 (6.5)	0.369	3 (6.2)	2 (10.0)	0.592
Conversion to open, n (%)	2 (5.0)	3 (8.6)	0.539	4 (10.0)	7 (11.3)	0.838	4 (8.3)	0	0.186
IWATE score, pt (SD)	3.5 (1.7)	4.4 (2.1)	0.052	4.1 (2.0)	4.4 (2.1)	0.452	5.5 (2.6)	6.4 (3.0)	0.179
Adjusted OT, min (SD)	222 (51)	258 (57)	**0.005**	252 (62)	258 (58)	0.659	301 (80)	315 (73)	0.506

(b) IWATE score	Learning phase	Post-learning	p	Learning phase	Post-learning	p	Learning phase	Post-learning	p
	n = 38	n = 37		n = 33	n = 69		n = 38	n = 30	
Overall morbidity, n (%)	10 (26.3)	3 (8.1)	**0.038**	10 (30.3)	13 (18.8)	0.197	6 (15.8)	8 (26.7)	0.274
Major morbidity (CD ≥ 3), n (%)	2 (5.3)	0	0.160	1 (3.0)	4 (5.8)	0.547	3 (7.9)	2 (6.7)	0.848
Conversion to open, n (%)	2 (5.3)	3 (8.1)	0.624	2 (6.1)	9 (13.0)	0.290	4 (10.5)	0	0.069
IWATE score, pt (SD)	3.4 (1.7)	4.5 (2.1)	**0.021**	3.8 (1.9)	4.5 (2.1)	0.089	5.3 (2.7)	6.3 (2.7)	0.148
Adjusted OT, min (SD)	221 (52)	257 (56)	**0.005**	246 (56)	260 (60)	0.244	297 (80)	316 (75)	0.321

Dichotomous variables are expressed as number with percentage. Continuous variables are expressed as mean with standard deviation (SD).

Significant values are given in bold.

When measuring the learning curve with the adjusted operative time, in the learning phase vs the post-learning phase respectively, the RobSug1 obtained a reduction in overall morbidity (25% vs 8.6%; p = 0.062) and major morbidity (5.0% vs 0; p = 0.183) together with an increase in the difficulty of the procedures [mean IWATE score (SD) of 3.5 (1.7) vs 4.4 (2.1); p = 0.052] although not reaching statistical significance. Conversions to open surgery remained stable (5.0% vs 8.6%; p = 0.539); the adjusted operative time was significantly longer [mean (SD) of 222 (51) vs 258 (57) min; p = 0.005]. For the laparoscopic procedures of the same surgeon (LapSurg1) in the learning phase vs the post-learning phase respectively, overall morbidity slightly decreased (27.5% vs 19.4%; p = 0.339) while major morbidity slightly increased (2.5% vs 6.5%; p = 0.369), conversions remained stable (10.0% vs 11.3%; p = 0.838) as well as the difficulty score [mean (SD) of 4.1 (2.0) vs 4.4 (2.1); p = 0.452] and the adjusted operative time [mean (SD) of 252 (62) vs 258 (58); p = 0.659]. In the control group (LapSurg2), overall and major morbidity increased (respectively 16.7% vs 30%; p = 0.219 and 6.2% vs 10%; p = 0.592), conversions did not occur in the post-learning phase (4.8% vs 0; p = 0.186), IWATE score [mean (SD) of 5.5 (2.6) vs 6.4 (3.0); p = 0.179] as well as the adjusted operative time [mean (SD) of 301 (80) vs 315 (73) min; p = 0.506] remained similar, none of the differences reached statistical significance (Table 4a).

When measuring the learning curve with the IWATE score, in the learning phase vs the post-learning phase respectively, the RobSurg1 drastically reduced overall and major morbidity (respectively 26.3% vs 8.1%; p = 0.038 and 5.3% vs 0; p = 0.160), conversions remained similar (5.3% vs 8.1%; p = 0.624), IWATE score significantly increased [mean (SD) of 3.4 (1.7) vs 4.5 (2.1); p = 0.021], adjusted operative time also increased [mean (SD) of 221 (52) vs 257 (56) min; p = 0.005]. For the laparoscopic procedures of the same surgeon (LapSurg1), overall morbidity decreased (30.3% vs 18.8%; p = 0.197) but major morbidity increased (3% vs 5.8%; p = 0.547) although both not reaching statistical significance, conversion rate doubled without showing statistical significance (6.1% vs 13.0%; p = 0.290), IWATE score slightly increased [mean (SD) of 3.8 (1.9) vs 4.5 (2.1); p = 0.089] and adjusted operative time remained similar [mean (SD) of 246 (56) vs 260 (60) min; p = 0.244]. In the control group (LapSurg2), overall and major morbidity were similar (respectively 15.8% vs 26.7%; p = 0.274 and 7.9% vs 6.7%; p = 0.848), conversion rate was nihil in the post-learning phase (10.5% vs 0; p = 0.069), IWATE score [mean (SD) of 5.3 (2.7) vs 6.3 (2.7); p = 0.148] and adjusted operative time [mean (SD) of 297 (80) vs 316 (75) min; p = 0.321] remained similar (Table 4b).

## Discussion

This study primarily investigated the learning process of RLR and LLR for a surgeon trained simultaneously in both MILS techniques and compared it to learning curve of LLR of another young consultant in otherwise similar conditions. The study, which is the first of its kind to our knowledge, found: (1) a substantial similarity of the progression in the robotic and laparoscopic technique for up to intermediate difficulty liver resections, countering the view of a faster learning curve for robotic surgery, and (2) the feasibility and safety of the simultaneous training in both MILS techniques at least for this type of procedures.

To measure the learning process of a surgical procedure is a challenging task^21^. Existing studies in the field of MILS are very heterogeneous because they use different types of statistical analysis or design, some include multiple-surgeons or institutional learning curves (sometimes not specifying the number of surgeons involved) rather than single-surgeon series, include a single standardized procedure (i.e., left lateral sectionectomy, or right hepatectomy, etc.) rather than the cumulative experience of different kinds of resections. Moreover, different variables can be used to estimate the learning curve (operative time, blood loss, conversions to open surgery, postoperative morbidity, progression in the difficulty score of MILS, etc.,)^11,14,22,23^. In summary, a consensus among clinicians is lacking regarding the optimal methodology to measure the LC, making it difficult to come to solid conclusions. A recent systematic review with meta-regression analysis by Chua, et al. queried the literature about the learning curve of MILS: out of 15 studies included for the quantitative analysis, 7 measured the learning curve of RLR. The number of cases needed to overcome the LC ranged from 11 to 30 but mixed anatomically minor and major hepatectomies^15^. The learning curve was measured with the CUSUM methodology in only 3 studies, was arbitrarily assessed in 3 others, finally one study identified the learning phase as the number of cases needed to obtain a significant increase in the difficulty score index. The variables of interest were also different: operative time was used in two studies, conversion to open surgery in two, blood loss and post-operative morbidity in two others, the difficulty score in one study. Moreover, three studies measured the learning curve of 2 surgeons while the remaining studies measured the learning curve of the whole team.

Data on the direct comparison of the learning curve of RLR vs LLR are scarce, but concordant in the sense of a shorter learning phase for RLR vs LLR. O’Connor, et al. in 2017 showed superior outcomes (blood loss, hospital stay and post-operative complications) for RLR vs LLR (minor hepatectomies) after 25 cases by two surgeons both already proficient in LLR. The cut-off of 25 cases however was arbitrability based on previous published papers on LLR and no CUSUM analysis was performed^24^. Efanov et al. defined the learning curve as the number of procedures necessary to significantly increase the difficulty index or the rate of resections in the posterior-superior liver segments^22,25^. The authors reported a two-times longer learning curve for LLR vs RLR (29 LLRs vs 16 RLRs) for two surgeons who initiated RLR shortly before LLR. The mean difficulty score of RLRs was 5.0 in the learning phase vs 7.3 (p < 0.001) once the learning curve was overcome (the last 24 cases). Of note, only 40 RLRs and 91 LLRs from two surgeons were included, peri-cystectomies were not excluded, cirrhotic patients were only 8% of RLR group and were all operated in the post-learning phase^22^. More recently, Krenzien et al. used a CUSUM analysis of the IWATE criteria as well as a “complexity-adjusted” CUSUM analysis of operative time, conversion rate and blood transfusions normalized by the IWATE score to compare the institutional LC of 132 RLRs vs 514 LLRs. The authors estimated the learning phase in 93 cases for RLR and 117 cases for LLR, about 4 times longer than reported in previous studies, but still shorter for robotics. In this case RLR program was developed after almost 10 years of LLR experience^16^.

Trying to capture a real-life learning curve of RLR and LLR, we decided to analyze all the consecutive MILSs performed by a single surgeon (single-surgeon learning curve), irrespective of the type of resection. The choice of a single-surgeon (rather than institutional) learning curve was because the robotic cases were performed by only one surgeon, as in most surgical realities starting robotic surgery programs, and was supported by the literature (14 out of 19 studies focused on single-surgeon learning curves in the recent meta-analysis by Chua, et al.)^15^. One could argue that our results may not be translated directly to realities where the robotic platform is shared among several liver surgeons. However, initial sharing of the robot is uncommon in new robotic programs.

When interpreting the learning curves of heterogeneous surgical procedures such liver resections, it is important to consider all the confounding factors (i.e., patient-, liver- or surgery-related) as well as the difficulty of the liver resection itself^17^. We used an analysis of covariance (ANCOVA) to estimate adjusted operative times. By using ANCOVA, we aimed to isolate and assess the effect of the independent variable (e.g., type of liver surgery, IWATE score, etc.) on the dependent variable (operative time) while simultaneously adjusting for the potential confounding effects of the covariates. In summary, adjusting implied that the estimated operative times took into account and controlled for the influence of relevant covariates, allowing to better understand the specific impact of covariates on the operative time. The estimation of the learning curve of the adjusted operative time has been recently adopted in robotic liver surgery and in other fields of robotic surgery such as upper gastrointestinal and hernia surgery^16,26,27^. The CUSUM methodology was then used to assess the learning phase, primarily considering the adjusted operative time, secondarily on conversions to open surgery, post-operative complications, and difficulty of MILS according to the IWATE criteria.

The interpretation of a learning curve is multifaceted, and this is a critical point in studies aiming at quantifying the problem. Typically, for simple surgeries, the operative time may be considered as the only relevant point, whereas for more difficult and heterogenous procedures, such as in liver surgery, the evaluation must also include complications, mortality, and the difficulty (IWATE score). Each of these indicators has advantages and drawbacks. The operative time depends on the surgeon's experience and on the complexity of a case, requiring the estimation of adjusted operative times. While complications and mortality are reliable endpoints, they are rare, and their assessment needs substantial sample sizes. The IWATE score is susceptible to selection bias compared to other CUSUM analyses, yet it is standardized and accepted in the field. We believe that the inclusion of all the indicators above (rather than just one of them) is an original aspect of our investigation. We could not clearly identify a sharp threshold in our series after which the adjusted operative times and complications dropped, nor a strong learning curve effect was demonstrated according to surgical outcomes of the pre- and post-learning phases. This was possibly due to the smooth learning curve effect that could be seen, on the CUSUM plots, after approximately 40 cases.

To further control the quality of the simultaneous training in RLR and LLR, we included a control group of laparoscopic procedures performed by a second surgeon only trained in LLR (LapSurg2) from another referral institution for HPB surgery. According to the CUSUM analysis of the adjusted operative time, the learning phase for LapSurg2 consisted in 48 cases and was indeed similar to that of the surgeon 1 in RLR and LLR (both 40 cases for RobSurg1 and LapSurg1). This taking also into account that in the LapSurg2 group there were more difficult cases (mean IWATE score of 4.0 for RobSurg1, 4.3 for LapSurg1 vs 5.8 for LapSurg2.

The finding of the similar learning curve with both techniques counters the common knowledge of a faster learning curve for RLR, reported in previous studies and for other surgical domains. In fact, the groups who reported a shorter learning curve for RLR vs LLR included surgeons with previous experience in LLR or did not clearly state whether the surgeons had a background of LLRs^15,16,24^. Our findings may be explained by the lack of a bias due to previous experience in former studies. One other possible reason is the absence of tools specific to liver resection by robotic approach which instead are available for the laparoscopic setting (contrary to other types of robotic surgery in which these are not needed).

The question of simultaneous training is now relevant, as the new generation of trainees in liver surgery is acceding to MILS. Major hepatobiliary centers usually started robotic programs after a solid background of laparoscopic liver surgery, and the point of the safety of simultaneous training could not be addressed. For example, D’Hondt and his team from Belgium performed LLRs-only for 8 years before the transition to RLR; similarly, the Berlin experience consisted of at least 7 years of LLRs before starting with RLRs^11,28^. The features of a purely robotic learning curve would be interesting but could not be addressed in the present study; however, it could be investigated following the same scheme.

Some limitations of this study must be addressed. First, this is a retrospective study of a single-surgeon learning curve including only anatomically minor or low-intermediate difficulty (IWATE) RLRs: our conclusions can’t be extrapolated to the training in robotics for more complex cases. Similarly, we were not able to comment on any differences between a concomitant (RLR and LLR) versus a sequential training (LLR followed by RLR) as the surgeon from the control group is only now starting the robotic training. Second, the control group (LapSurg2) consisted of more difficult resections compared to the procedures from the Surgeon1 (RobSurg1, LapSurg1, concomitant training) resulting in la slightly longer learning phase. Conversely, the incidence of underlying chronic liver disease (notably cirrhosis) and portal hypertension rate were significantly lower in the control group. We believe this did not bias our conclusions as our primary focus was on the simultaneous training cohorts, which consisted of similar procedures (as per difficulty score) in similar populations. Third, despite MILS cases in the cohorts of simultaneous training were selected in principle to be similar (patients characteristics, IWATE score), a selection bias could not be completely excluded because of the retrospective nature of the analysis and the fact that the robotic platform was available only once a week or once every two weeks, while laparoscopy was freely available. This fact could have led to a more cautious selection of cases for RLRs. The ANCOVA test was adopted to mitigate all these confounding factors. Last, the learning curve effect can be visually estimated on CUSUM charts, but its interpretation may not be univocal. Therefore, we carried out further CUSUM analyses for conversions to open surgery, post-operative morbidity and increase in the IWATE difficulty score. We could not find a “learning curve effect” in the LapSurg1 and LapSurg2 groups as per postoperative complications and conversions. We hypothesized that this was because of the clinically appropriate selection bias, choosing easier cases for the beginning and progressing carefully to more difficult resections. Accordingly, we were not able to comment on the learning curve obtained from the CUSUM analyses of these variables.

In conclusion, our experience showed a similar learning phase of approximately 40 cases for simultaneous training up to intermediate difficulty RLRs and LLRs. The concomitant training in both techniques was safe in the development of a MILS program. Our findings tend to counter the view of a faster learning curve for robotic liver resections (at least for low and intermediate difficulty procedures), possibly because of the lack of a bias due to previous experience in former studies, and because of the absence of dedicated robotic tools for parenchymal dissection.

### Supplementary Information

Supplementary Figure 1.

## Data Availability

The dataset analyzed during the current study is available from the corresponding author on request.
