# Supplementary material for: Concomitant training in robotic and laparoscopic liver resections of low-to-intermediate difficulty score: a retrospective analysis of the learning curve
Source: Sci Rep. 2024 Feb 13;14:3595. doi: 10.1038/s41598-024-54253-z (PMC10864263; doi:10.1038/s41598-024-54253-z)
Supplement: Supplementary file 1 — Supplementary Figure 1. [file 41598_2024_54253_MOESM1_ESM.docx]

Supplementary material:


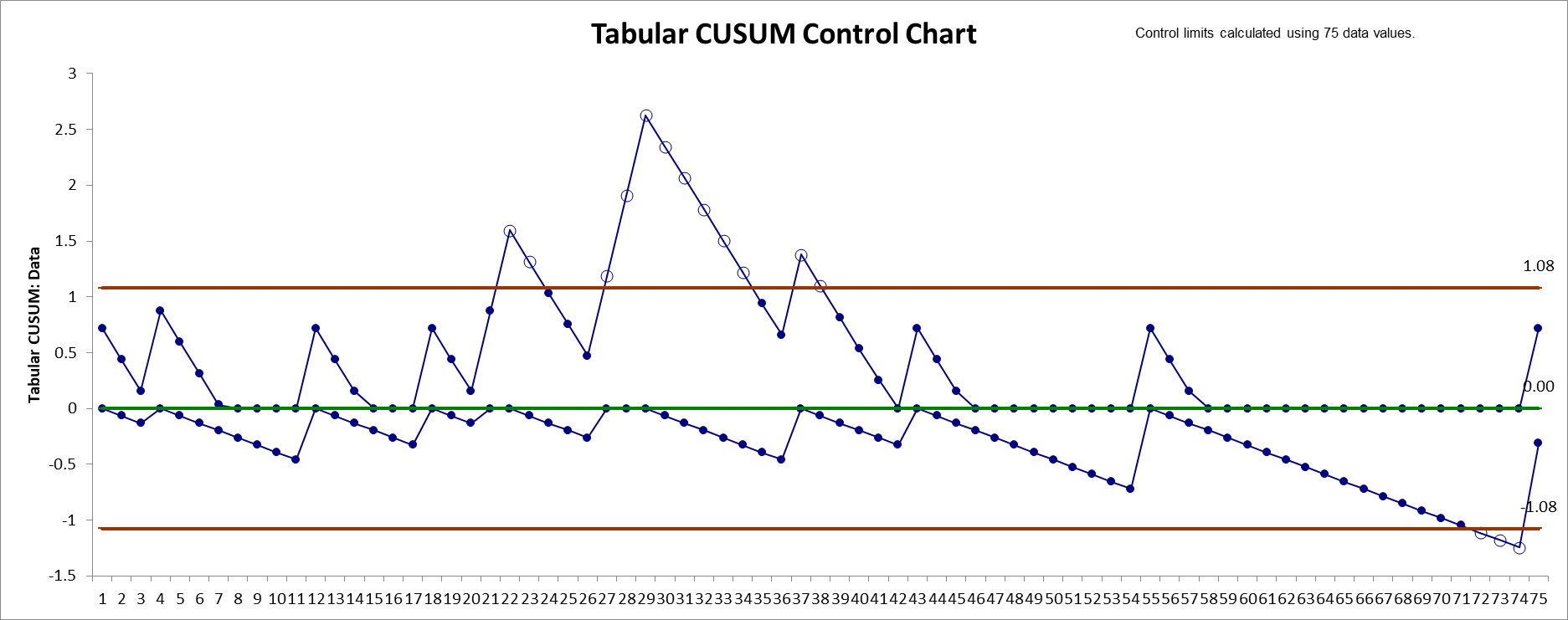


Table S1: The CUSUM analysis of post-operative morbidity. After a learning phase of 42 cases, the postoperative morbidity of RLRs (RobSurg1) plateaued.

Figure S1: The CUSUM analysis of post-operative morbidity. After a learning phase of 42 cases, the post-operative morbidity of RLRs (RobSurg1) plateaued.
